# *Bombyx mori* Silk Fibroin Scaffolds with *Antheraea pernyi* Silk Fibroin Micro/Nano Fibers for Promoting EA. hy926 Cell Proliferation

**DOI:** 10.3390/ma10101153

**Published:** 2017-10-03

**Authors:** Yongchun Chen, Weichao Yang, Weiwei Wang, Min Zhang, Mingzhong Li

**Affiliations:** National Engineering Laboratory for Modern Silk, College of Textile and Clothing Engineering, Soochow University, No. 199 Ren’ai Road, Industrial Park, Suzhou 215123, Jiangsu, China; 20155215014@stu.suda.edu.cn (Y.C.); lerui27@tom.com (W.Y.); 20154215028@stu.suda.edu.cn (W.W.); 20154215027@stu.suda.edu.cn (M.Z.)

**Keywords:** scaffolds, silk fibroin, electrospinning, lyophilization

## Abstract

Achieving a high number of inter-pore channels and a nanofibrous structure similar to that of the extracellular matrix remains a challenge in the preparation of *Bombyx mori* silk fibroin (BSF) scaffolds for tissue engineering. In this study, *Antheraea pernyi* silk fibroin (ASF) micro/nano fibers with an average diameter of 324 nm were fabricated by electrospinning from an 8 wt % ASF solution in hexafluoroisopropanol. The electrospun fibers were cut into short fibers (~0.5 mm) and then dispersed in BSF solution. Next, BSF scaffolds with ASF micro/nano fibers were prepared by lyophilization. Scanning electron microscope images clearly showed connected channels between macropores after the addition of ASF micro/nano fibers; meanwhile, micro/nano fibers and micropores could be clearly observed on the pore walls. The results of in vitro cultures of human umbilical vein endothelial cells (EA. hy926) on BSF scaffolds showed that fibrous BSF scaffolds containing 150% ASF fibers significantly promoted cell proliferation during the initial stage.

## 1. Introduction

Establishing microcirculation within a three-dimensional scaffold is the key challenge in the field of tissue engineering and situ tissue regeneration, as a microvessel capillary network needs to be generated in the scaffold as soon as possible [1,2,3]. Utilizing the micro/nano fiber cell-contact guidance effect, and endowing scaffolds with a micro/nano structure that mimics the extracellular matrix (ECM), but with more inter-pore channels, thereby providing physical stimulation signals and a suitable microenvironment for endothelial cell adhesion, proliferation and guide cell migration, have been found to be effective ways to achieve rapid scaffold neovascularization [4,5,6].

Silk proteins are natural fibrous proteins that have been widely used in the field of tissue engineering because of their biocompatibility and biodegradability. *Antheraea pernyi* silk fibroin (ASF) and *Bombyx mori* silk fibroin (BSF) are fibroins from two of the most widely used species of silkworms [7,8,9,10,11,12,13]. Porous BSF scaffolds have been applied in the regeneration of various tissues such as skin, blood vessels, bone and ligaments [14,15,16,17]. The structure of ASF is clearly different from that of BSF. In contrast to the amino acid composition of BSF, each H chain of ASF contains 12 Arg-Gly-Asp (RGD) tripeptide sequences, which serve as specific adhesion sequences for mammalian cells [18,19,20,21]. There are several methods of preparing three-dimensional porous BSF scaffolds, including the lyophilization, freeze-thaw, salt-leaching and 3D-printing methods [22,23,24,25,26,27]. These approaches are powerful for porous structure fabrication, but the products lack sufficient inter-pore channels and 3D nanofibrous structure. Consequently, although porous BSF scaffolds provide 3D microscopic configurations for tissue constructs, the pore walls of the scaffolds provide only a two-dimensional growth environment for cells. Therefore, the design of BSF scaffolds with a fibrous microstructure is important for further improvement of their biomimeticity. Electrospinning, as a nanofiber fabrication technique, offers several advantages, such as a simple and versatile procedure, and the ability to prepare nanofibers of very small diameter [28,29]. Unfortunately, in silk-based scaffolds, it is difficult to use current electrospinning techniques to prepare complex 3D nanofibrous BSF scaffolds with adjustable pore size. Therefore, achieving more inter-pore channels and a nanofibrous network with a suitable pore size remains a challenge in the fabrication of 3D BSF scaffolds.

Porous BSF scaffolds with controllable porosity pore size and biodegradability can be prepared by the freeze-drying method [30,31,32,33]. We hypothesized that electrospun ASF fibers could be inlaid in the surface of pore walls by the dispersion of short ASF fibers in BSF solutions followed by freeze-drying, producing a fibrous BSF scaffold that is more favorable for cell adhesion and proliferation than a no-fiber BSF scaffold. The aim of this study was to inlay micro/nano fibers in the pore walls of 3D BSF scaffolds and improve the connection between the macropores, thereby enhancing cell adhesion, proliferation, vascularization and tissue regeneration when the scaffolds are used for in situ tissue regeneration or tissue engineering. Based on our hypothesis, in this study, we used a facile electrospinning method to produce ASF fibers with an average diameter of 324 nm. These electrospun fibers were cut into short fibers (~0.5 mm) and then dispersed in a BSF solution. Next, BSF scaffolds containing ASF fibers were prepared by lyophilization. Human umbilical vein endothelial cells (EA. hy926) were cultured on the fibrous BSF scaffolds to evaluate the ability of the scaffolds to promote cell proliferation.

## 2. Results

### 2.1. Morphology of ASF Micro/Nano Fibers

**Figure 1:**
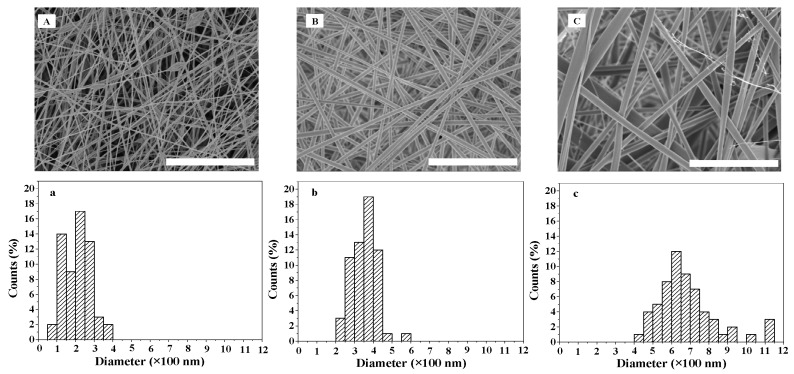
SEM images and diameter distribution of ASF fibers at different electrospinning concentrations: (**A**,**a**) 7 wt %; (**B**,**b**) 8 wt %; (**C**,**c**) 9 wt %. Scale bars: 10 µm.

Spinning concentration significantly affects the diameter and distribution of fibers [34]. The SEM images and diameter distribution of ASF micro/nano fibers at different spinning concentrations are shown in Figure 1. At a concentration of 7 wt %, fibers with beads were observed. The beads were irregularly distributed, and the bead diameter was approximately 0.6~1.2 µm. The fiber diameter size was distributed between 50 and 400 nm, and the most common fiber diameter range was 200~250 nm (Figure 1A,a). Uniform and bead-free fibers with an average diameter of 324 nm were obtained with an 8 wt % ASF solution. The distribution of fiber diameter size was between 200 and 600 nm, and the most common fiber diameter range was 350~400 nm (Figure 1B,b). Further increasing the ASF concentration to 9 wt % resulted in bead-free fibers with a broader diameter distribution, ranging between 400 and 1150 nm, within which the most common fiber diameter range was 600~650 nm(Figure 1C,c).

### 2.2. Morphology of BSF Scaffolds Containing ASF Micro/Nano Fibers

**Figure 2:**
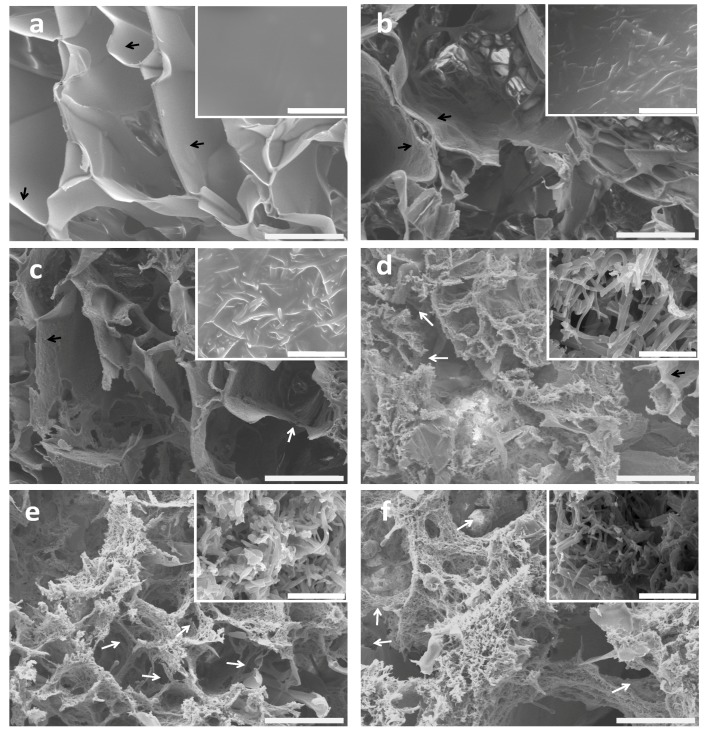
SEM images of BSF scaffolds with different proportions of ASF micro/nano fibers added: (**a**) 0; (**b**) 50%; (**c**) 100%; (**d**) 150%; (**e**) 200%; (**f**) 250%. The insert images show high magnification of the porous walls of the scaffolds. The lamellar pore walls and connected channels are indicated by black and white arrows, respectively. Scale bars: (**a**–**f**) 100 µm; inset images 10 µm.

The fiber proportions affected the morphology and microstructure of the BSF scaffolds. Figure 2 shows the effects of different ASF fiber proportions on the microstructure and topography of the BSF scaffolds. The no-fiber BSF scaffolds showed a macroporous structure with pore diameters of approximately 100 to 300 µm and smooth pore wall surfaces (Figure 2a). Marked differences were observed in the BSF scaffolds containing ASF fibers. When 50% (*w*/*w*) ASF fibers were added to the BSF solution, micropores with sizes of several microns and irregular ASF fibers were observed on the pore walls after lyophilization (Figure 2b). When the proportion of ASF fibers was increased to 100% of the amount of BSF, parts of the pore walls were observed to consist of ASF fibers, and the number of micropores, whose sizes ranged from several microns to 50 µm, clearly increased. A small number of inter-pore channels were observed (Figure 2c). When the proportion of ASF fibers was increased to 150%, most of the pore walls were observed to consist of fibers, with pore sizes ranging from several microns to 30 µm, and more connected channels between the macropores were observed (Figure 2d). Further increasing the proportion of ASF fibers to 200% or more could cause the lamellar pore walls to disappear. ASF fibers could only be observed on the pore walls, and inter-pore channels could be clearly observed (Figure 2e,f).

### 2.3. In Vitro Cell Growth within BSF Scaffolds

**Figure 3:**
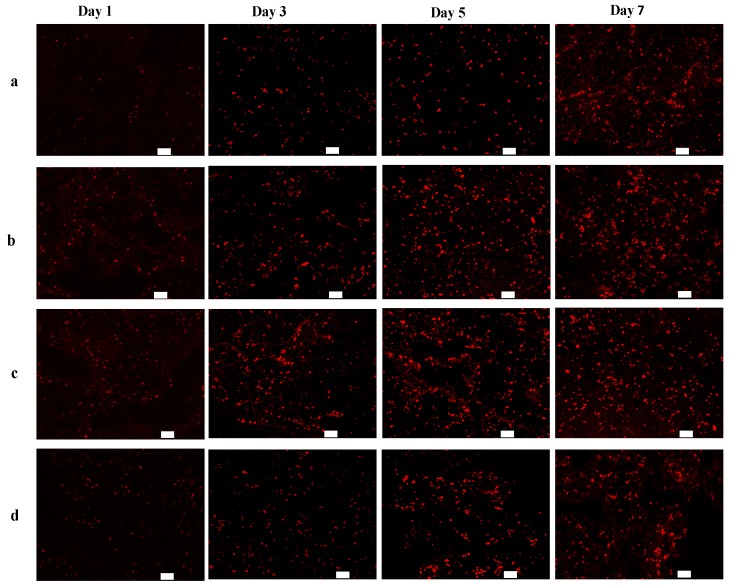
Fluorescence microscope images of CM-Dil stained BSF scaffolds seeded with EA. hy926 cells on days 1, 3, 5 and 7. Proportions of ASF micro/nano fibers: (**a**) 0; (**b**) 50%; (**c**) 150%; (**d**) 250%. Scale bar: 100 µm.

**Figure 4:**
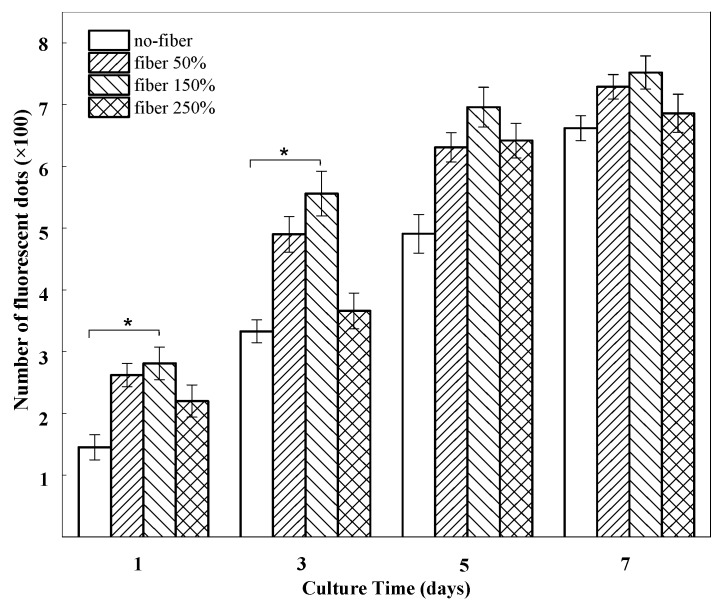
The number of fluorescent dots on BSF scaffolds with different proportions of ASF micro/nano fibers at days 1, 3, 5, 7. Error bars represent mean standard deviation with *n* = 3 (* *p* < 0.05).

The potential effect of the BSF scaffolds on EA. hy926 cell growth was determined in vitro. Figure 3 presents confocal laser scanning microscopy (CLSM) images showing the growth of EA. hy926 cells (red) on different BSF scaffolds. After 1 day of culture, the cells were distributed sparsely on all scaffolds. On day 3, the numbers of living cells on the fibrous BSF scaffolds containing 50% and 150% ASF micro/nano fibers were clearly greater than those on other BSF scaffolds, as shown by the CLSM images (Figure 3b,c). After 5 and 7 days of culture, the red fluorescence density levels of the fibrous BSF scaffolds containing 50% and 150% ASF micro/nano fibers were greater than those of no-fiber scaffolds (Figure 3a–c). Quantitatively, the numbers of fluorescent dots on BSF fibrous scaffolds containing 50% and 150% ASF micro/nano fibers were greater than those of no-fiber scaffolds after culturing for 1 and 3 days. On day 5 and day 7, the numbers of fluorescent dots on BSF fibrous scaffolds containing 50% and 150% ASF micro/nano fibers were still greater than those in no-fiber scaffolds, but the differences were not significant (Figure 4).

**Figure 5:**
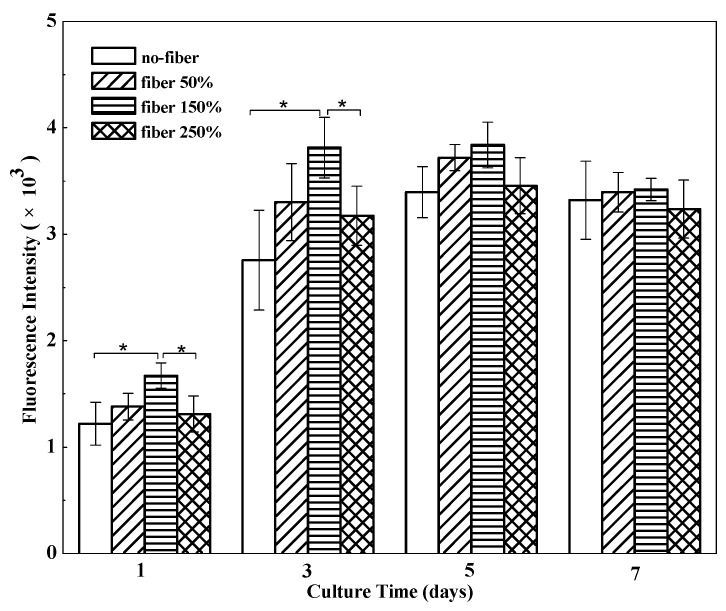
Proliferation of EA. hy926 cells cultured on BSF scaffolds with different proportions of ASF micro/nano fibers at days 1, 3, 5, 7. Error bars represent mean standard deviation with *n* = 3 (* *p* < 0.05).

To assess cell proliferation on different BSF scaffolds, the Alamar Blue assay was performed (Figure 5). EA. hy926 cells were cultured to measure cell proliferation on different scaffolds. Compared to that in no-fiber BSF scaffolds, the number of living cells in the fibrous BSF scaffolds increased faster from day 1 to day 3. Especially in fibrous BSF scaffolds with 150% ASF micro/nano fiber, the total cell number increased rapidly, and the cell number was significantly higher than the numbers on other scaffolds (*p* < 0.05) after culturing for 3 days, suggesting that the fibrous BSF scaffolds provided a more favorable environment for the proliferation of EA. hy926 cells. After 5 and 7 days of culture, the cell numbers in fibrous BSF scaffolds were still greater than those in no-fiber scaffolds, but the differences were not significant (*p* > 0.05), which is in agreement with the results shown in Figure 3 and Figure 4.

## 3. Discussion

**Figure 6:**
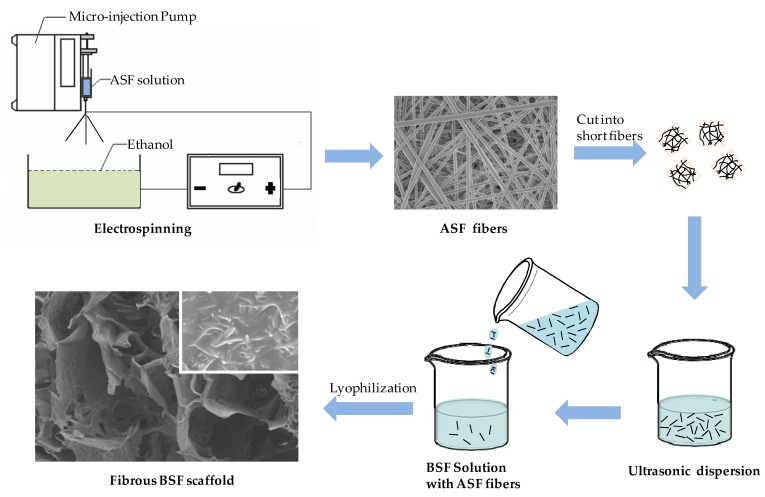
Schematic process of preparation for fibrousBSF scaffolds using freeze-drying method.

BSF scaffolds were endowed with a microstructure resembling that of an ECM, which was an effective approach to improving cell migration and proliferation within scaffolds, because the fine micro/nano fibrous network provides more physical sites and physical stimulus signals for cell attachment and spreading [2,35]. BSF scaffolds have been fabricated by various methods, such as gas-foaming, freeze-drying and salt-leaching, and silk fibroin nanofibers can be prepared by electrospinning or self-assembly [29,36]. However, most of these methods cannot produce BSF scaffolds with nanofibrous structures and a high number of connected channels between macropores. Herein, an ASF solution with a concentration of 8 wt % was used to prepare ASF fibers by electrospinning, and the resulting ASF fibers were dispersed in a BSF solution. Then, the fibers were successfully integrated into the pore walls of BSF scaffolds fabricated by the freeze-drying method (Figure 6). In recent years, research on the preparation techniques [37], structural control methods [32], physical properties [38], cell biocompatibility and biodegradability [11,39] of BSF scaffolds, which were used as the tissue regeneration template, has been more extensive than that on ASF scaffolds. From the perspective of application, it is possible to obtain more experimental data support for BSF scaffolds than for ASF scaffolds; hence, BSF was used as the base material for the scaffolds in this study. The structure of the fibrous BSF scaffolds enhanced nutrient and waste transfer, and the ASF fibers on/in the pore walls of the scaffolds provided a contact guidance signal for cell attachment and migration, thus promoting cell proliferation within the BSF scaffolds.

As shown by Deitzel et al. [40], increasing the concentration of polymer solution can lead to higher solution viscosity and lower tension. These properties allowed the solutions to be sprayed stably and continuously from the spinneret, and the jet was then elongated and drawn into a smooth fiber with a large diameter under electrostatic stresses (Figure 1C,c). The fibers electrospun from an 8 wt % ASF solution exhibited an average diameter of 324 nm, with the most common diameter range being from 350 to 400 nm (Figure 1b). At a concentration of 7 wt %, fibers with beads could be clearly observed in the formed scaffolds, but could not be clearly observed at concentrations of 8% and 9%. The beads were irregularly distributed, and the bead diameter was approximately 0.6~1.2 µm (Figure 1a). It has been reported that a critical concentration of polymer solution needs to be exceeded for electrospinning, because, below such a concentration, chain entanglements of the polymer are insufficient to stabilize the jet, and a bead-like structure tends to form in the fiber [41].

Recently, natural BSF fibers were incorporated into porous BSF scaffolds to improve tissue reconstruction [42,43]. However, due to their large diameter, incorporated BSF fibers have not been fully adapted to individual cell guidance in tissue regeneration [44]. Given the above drawbacks, we added electrospun ASF micro/nano fibers to the BSF solution to integrate ASF fibers into the pore walls during BSF scaffold formation. It has been reported that the directional ice freezing and the addition of hydroxyapatite could affect the structural morphology of the BSF scaffolds [45,46]. In our study, we found that the addition of ASF fibers significantly affected the structural morphology of the BSF scaffolds. After different proportions of ASF fibers were added to the BSF solution, obvious changes could be observed in the structural morphology of the pores. Some micropores with sizes ranging from microns to tens of microns appeared in the pore walls, and connected channels between macropores could be clearly observed on the pore walls (Figure 2b–d). When the proportion of ASF fibers reached 200% of the quantity of BSF, the pore wall consisted of ASF micro/nano fibers, and a large number of micropores ranging from microns to tens of microns in diameter were present in the pore wall; moreover, the connected channels between macropores could be more clearly observed in the pore walls (Figure 2f).

Compared with no-fiber scaffolds, fibrous scaffolds may provide more adhesion sites for cells. It is known that filopodia adhere first to nanofibrils on a patterned surface. Therefore, cells preferentially adhere to nanofibrillar islands instead of to smooth surfaces [47,48]. In fibrous BSF scaffolds, the micro/nano fibers on the surfaces of the pore walls and the connected channels between macropores are able to provide a large surface area, high level of physical stimulation, and a large number of sites for cell proliferation [49]; therefore, the number of living cells on the fibrous BSF scaffolds was clearly greater than that on the no-fiber BSF scaffolds (Figure 3b,c and Figure 4). Furthermore, ASF micro/nano fibers and the connected channels between macropores within the scaffolds may provide passageways for cell extension and migration. Due to their increased surface area, cells seeded on fibrous BSF scaffolds proliferated much faster than those on no-fiber scaffolds after 3 and 5 days of culture (Figure 3b,c and Figure 4). The cell proliferation results indicated that BSF scaffolds containing ASF fibers promoted EA. hy926 cell proliferation after 1 day and 3 days of culture (Figure 5). This effect clearly suggests that the microstructure within the scaffolds plays a key role in initial cell proliferation. The densities of cells seeded on scaffolds in our experiment were 1 × 10^5^ cells/well for cell proliferation, as has been commonly used in the literatures [50,51]. After 5 and 7 days of culture, the cell proliferation rate on different BSF scaffolds showed no significant difference, so if differences in cell proliferation on the BSF scaffolds beyond 3 days are detected, the densities of cells seeded on scaffolds should be less than 1 × 10^5^ cells/well. A similar phenomenon was also observed on the BSF/wollastonite composite scaffolds [52].

## 4. Materials and Methods

### 4.1. Preparation of BSF Solution

Regenerated BSF solution was prepared according to our previously published procedures [35]. Briefly, *Bombyx mori* raw silk fibers (Huzhou, China) were boiled three times in a 0.05% Na_2_CO_3_ solution for 30 min to remove sericin and dried at 60 °C after thorough rinsing. The degummed fibroin was dissolved in a 9.3 M LiBr solution at 60 ± 2 °C for 1 h. A 4 wt % regenerated BSF solution was obtained after dialysis (MWCO 9–12kDa) in deionized water for 4 days.

### 4.2. Preparation of ASF Solution and Solids

*Antheraea pernyi* raw silks (Dandong, China) were degummed by three treatments with 0.25% Na_2_CO_3_ aqueous solution at 98 °C–100 °C for 30 min. After being rinsed and dried at 60 °C, the degummed ASF fibers were dissolved in a melted Ca(NO_3_)_2_·4H_2_O solution at a 1:10 (*w*/*v*) bath ratio for 5 h at 105 °C. The cooled solution was dialyzed (MWCO 9–12kDa) against deionized water for 4 days and then filtered and lyophilized to obtain the regenerated ASF solids. Finally, the ASF solids were placed in −18 °C refrigerator for reserve.

### 4.3. Electrospinning of ASF Fibers

ASF solutions (7, 8, 9 wt %) were prepared by dissolving ASF solids in hexafluoroisopropanol (HFIP; Sigma-Aldrich, St. Louis., MO, USA) and stirring for 3 h at room temperature. Electrospinning was performed at a flow rate of 0.3 mL/h under a 12 kV voltage field, and the distance between the needle tip and the grounded target was 10 cm. The electrospun fibers were deposited in an ethanol solution. The ASF micro/nano fibers were electrospun for 3 h and then dried in vacuo at room temperature for 24 h. In this study, the ASF solids were prepared from the same ASF solution at the same time after lyophilization.

### 4.4. Preparation of BSF Scaffolds Containing ASF Micro/Nano Fibers

Figure 6 shows the process of preparation for fibrous BSF scaffolds using the freeze-drying method. The ASF fibers were cut into short fibers (~0.5 mm) and sonicated at 40% power using a Bioruptor sonicator (JYD-650L, Shanghai Zhixin instrument Co., Ltd., Shanghai, China) to achieve dispersion, and then added slowly to the BSF solution at proportions of 0, 50, 100, 150, 200 and 250% of the BSF weight in solution. Next, polyethylene glycol diglycidyl ether (PEG-DE; Sigma-Aldrich, St. Louis, MO, USA) was added slowly to this mixture while stirring to obtain a final PEG-DE: BSF ratio of 0.3:1 (*w*/*w*), and then the solution was poured into stainless steel vessels. The vessels were placed at −40 °C for 24 h to freeze the samples, and the samples were lyophilized for approximately 48 h to obtain porous BSF scaffolds containing ASF micro/nano fibers, termed “fibrous BSF scaffolds”. Here, the BSF solution was prepared from the same solution at the same time.

### 4.5. Scanning Electron Microscopy

The cross-sectional morphology of the scaffolds and ASF fibers was observed using a scanning electron microscope (SEM; S-4800, Hitachi, Tokyo, Japan). Samples were mounted on a copper plate and sputter-coated with a gold layer 20–30 nm in thickness prior to imaging. The diameter of the fibers was analyzed based on the SEM images using the Nano Measurer analysis software (Department of Chemistry, Fudan University, Shanghai, China). The fiber diameters of each group were measured for a total of 100 fibers.

### 4.6. Cell Culture and Observation

The BSF scaffolds were cut into discs 1.5 cm in diameter and immersed in 75% ethanol for 15 min. Sterilized samples were then placed in 24-well plates and washed three times with sterile phosphate-buffered saline (PBS; 0.1 M, pH 7.4) for cell seeding. Human umbilical vein endothelial cells (EA. hy926 cells; ATCC, Rockefeller, Maryland, USA) were cultured in Dulbecco’s modified Eagle medium (DMEM; HyClone, Logan, UT, USA) supplemented with 10% fetal bovine serum (FBS; HyClone, Logan, UT, USA) and 1% streptomycin-penicillin (Beyotime, Nantong, China). The medium was replaced every 2 days, and the cell cultures were maintained in a humidified incubator at 37 °C and 5% CO_2_. After reaching 80% confluence, the cells were detached from the Petri dish and washed three times with PBS. The cells were then adjusted to a concentration of 1 × 10^6^ cells/mL. Next, we added Cell-Tracker™ CM-Dil (Invitrogen, Carlsbad, CA, USA), a membrane dye and can label more than 95% of the treated cells in the cell suspension; the diluted concentration of CM-Dil was 5 μg/mL. Next, we incubated the cells at 4 °C for 15 min and incubated the cells in a 37 °C humidified incubator for 10 min, and then we washed the cells twice with PBS. Finally, the cells were seeded into the scaffolds containing 0, 50, 150 and 250% ASF micro/nano fibers at a density of 1 × 10^5^ cells/well and incubated in a humidified incubator at 37 °C and 5% CO_2_ for 4 h to allow them to adhere to the scaffold before the addition of 1 mL complete medium to each well. The cell-seeded scaffolds were replenished with fresh medium every 2 days. After culturing for 1, 3, 5 and 7 days, the cell-seeded scaffolds were washed three times with PBS before confocal laser scanning microscopy (CLSM; IX81/FV1000, Olympus, Tokyo, Japan) observation using a laser beam at a wavelength of 550 nm, all quantitative results of fluorescent dots were obtained from three different horizons (1.2 × 1.2 μm) for analysis.

### 4.7. Cell Proliferation

Cell viability and proliferation were assessed by the Alamar Blue assay following the vendor’s instructions [53]. Briefly, cells on scaffolds containing 0, 50, 150 and 250% ASF micro/nano fibers were incubated in medium supplemented with 10% (*v*/*v*) Alamar Blue fluorescent dye (Biosource, Camarillo, CA, USA) for 2 h (*n* = 3). One hundred microliters of medium from each example was read at 560/590 nm in a Synergy HT Multi-Mode Microplate Reader (Bio-Tek Instruments, Winooski, VT, USA). Medium supplemented with 10% Alamar Blue dye was used as a negative control and its fluorescence intensity was subtracted from that of the experimental groups containing cells.

### 4.8. Statistical Analysis

All assays were repeated with a minimum of n = 3. Data were analyzed using SPSS 16.0 software for Windows, student version. Statistically significant values were defined as *p* < 0.05 based on one-way analysis of variance (ANOVA).

## 5. Conclusions

Novel BSF scaffolds containing ASF micro/nano fibers and inter-pore channels were fabricated by adding ASF micro/nano fibers to a BSF solution and then freeze-drying. The addition of ASF micro/nano fibers generated a large number of micropores and micro/nano fibers on/in the pore walls of the BSF scaffolds, dramatically increasing the number of interconnected channels between the macropores. In vitro cell culture experiments demonstrated that fibrous scaffolds containing 150% ASF fibers can significantly promote EA. hy926 cell proliferation during the initial stage.
